# Tweets That Matter: Exploring the Solutions to Maternal Mortality in the United States Discussed by Advocacy Organizations on Twitter

**DOI:** 10.3390/ijerph20095617

**Published:** 2023-04-24

**Authors:** Diane Ezeh Aruah, Yvonne Henshaw, Kim Walsh-Childers

**Affiliations:** 1 Communication Department, Tennessee State University, Nashville, TN 37209, USA; 2 Air Liquide, St Ne N20, Calgary, AB T2E 7H7, Canada; yvonne.henshaw@airliquide.com; 3 College of Journalism and Communication, University of Florida, Gainesville, FL 32611, USA; kimwc@ufl.edu

**Keywords:** maternal health, maternal mortality, birth equity, twitter, advocacy, United States

## Abstract

This study investigated maternal mortality solutions mentioned on Twitter by maternal health advocacy organizations in the United States. Using qualitative content analysis, we examined tweets from 20 advocacy organizations and found that the majority of the tweets focused on policy, healthcare, community, and individual solutions. The most tweeted policy solutions include tweets advocating signing birth equity, paid family leave, Medicaid expansion, and reproductive justice bills, whereas the most tweeted community solutions were funding community organizations, hiring community doulas, and building community health centers. The most tweeted individual solutions were storytelling, self-advocacy, and self-care. These findings provide insights into the perspectives and priorities of advocacy organizations working to address maternal mortality in the United States and can inform future efforts to combat this critical public health issue.

## 1. Introduction

The World Health Organization (WHO) defines maternal mortality as the death of a woman while pregnant or within 42 days of termination of pregnancy, irrespective of the duration or site of the pregnancy, from any cause related to or aggravated by the pregnancy or its management [1]. The maternal mortality rate in the United States has for many years exceeded that of other high-income countries [2]. The Centers for Disease Control and Disease Prevention (CDC) reported that the maternal mortality rate rose in 2021 to 32.9 deaths per 100,000 live births, compared with a rate of 23.8 in 2020 and 20.1 in 2019. In 2021, the maternal mortality rate for Black women was 69.9 deaths per 100,000 live births, 2.6 times higher for White women [3]. However, in the Netherlands, Norway, and New Zealand, the rate dropped to three or fewer women per 100,000 [4]. 

Several governmental and non-governmental organizations, including the US Health and Human Services, are working tirelessly to raise awareness of the causes of maternal mortality in the United States, including racism and discrimination, socioeconomic determinants of health, chronic conditions, and limited access to healthcare in rural communities [5]. Maternal health advocacy groups have turned to social media, especially Twitter, to promote maternal health and raise awareness about maternal mortality trends [6]. Previous studies have investigated the use of social media to create maternal health awareness [7,8]. However, few studies have examined the use of Twitter to advocate for increased attention to maternal health issues [6]. No study has specifically explored the solutions these organizations advocate for and how these solutions could potentially impact public opinion, discourse, and political actions. This study seeks to fill this gap by conducting a qualitative content analytical study on how maternal health organizations use Twitter to promote maternal mortality reduction in the United States. Specifically, this study investigates the categories of solutions these maternal health groups promote. By analyzing a sample of tweets from the groups, this study provides a deeper understanding of the potential role of social media in spreading awareness about maternal health solutions and influencing the public and policymakers to take necessary action to combat the maternal mortality crisis in the United States.

### 1.1. Twitter as a Vital Tool for Public Health Advocacy

With more than 450 million users, Twitter is one of the most effective digital tools for raising awareness about public health issues in the United States and globally [9,10]. Twitter provides a context for billions of users to connect, express sentiments, and provide in-the-moment status updates [11,12]. Twitter has been extensively used for health information sharing, primary care, delivery of health support, primary prevention, and public health education [13,14]. For example, health organizations have used Twitter to spread information about the COVID-19 pandemic, contagious disease outbreaks, tobacco, and influenza [14].

Twitter has several features that make it a valuable tool for health advocacy. Twitter has a large user base, which allows advocates to reach a broad audience quickly and easily. The ability of users to retweet and follow advocacy groups fosters user engagement [15,16]. Twitter’s use of hashtags makes it simpler for health advocates to categorize and organize their messages and for users to find relevant information on specific health topics. Twitter is prominent for its real-time updates, which is particularly essential for health advocates during public health emergencies, as it allows for the timely dissemination of critical information [16]. The reply and tag functions of Twitter enable users to engage and build relationships with users, which can be beneficial for promoting healthy behaviors and building awareness of health issues [16]. Twitter’s analytical tools allow users to measure the impact of their messages, track user engagement, and identify trends and patterns in health-related conversations. Twitter’s hyperlinks can encourage followers to organizations’ blogs or other social media sites [17]. The multimedia feature of Twitter allows health organizations to disseminate health tips and medical information through photos and videos, thus increasing the audience’s perceived value of the content [18].

Twitter can be used to launch health campaigns, raise funds for health causes and generate public support for policy changes that can improve health outcomes. In addition, Twitter allows health organizations lacking financial resources and staffing to promote their health campaigns limitlessly [19]. Twitter serves as an agenda-setter for health advocacy groups [20]. Advocacy groups consistently post helpful information and health tips to influence the public agenda regarding the most critical health issues [21]. Using Twitter to raise awareness, advocacy groups can pressure politicians and health professionals to act and change policies [22].

### 1.2. Maternal Mortality Advocacy in the United States

Even before the COVID-19 pandemic, maternal mortality advocacy efforts have gained significant momentum in the United States media [23]. Advocacy organizations such as the Black Mamas Matter Alliance, March for Moms, and Every Mother Counts have utilized Twitter to post information about trends and news about maternal health issues in the United States. They aim to improve access to high-quality maternal care, address systemic racism in healthcare, and advocate for policies that support maternal health [24].

One of the critical issues maternal health advocates are addressing is the lack of access to high-quality maternal care, particularly in Black communities [6,25]. More than 50% of US counties are without a practicing obstetrician, and many rural hospitals have closed their obstetric units due to financial challenges [26]. This lack of access to care is hazardous for women with high-risk pregnancies or underlying health conditions. To address this issue, maternal health advocates call for increased funding for maternal health programs, services, and policies supporting the recruitment and retention of obstetric providers in underserved areas. For example, The Black Mamas Matter Alliance is a national voice and coordinating entity for stakeholders advancing maternal health, rights, and justice [27]. These organizations provide technical assistance, training, and capacity building for grassroots organizations and provide housing, employment, education, doula care, and mental health support [27].

Maternal health advocates also address the impact of systemic racism on maternal health outcomes [28]. Research has shown that Black and indigenous women are more likely to experience racism and discrimination in healthcare settings, leading to high disparities in maternal health outcomes [29]. This includes poor access to high-quality care, provider bias and stereotyping, and inadequate pain management during childbirth [30]. To address this issue, advocates work to train healthcare providers to practice culturally competent care, increase the diversity of the healthcare workforce and address biases in medical research and treatment guidelines [31]. Maternal health advocates also address the need for policy changes that support maternal health [32]. This includes policies that provide paid family leave, affordable childcare, and access to mental health care. The National Partnership for Women and Families, for example, advocates for the establishment of a federal paid leave program that provides at least 12 weeks of paid leave for all workers, thus allowing new mothers to take time to recover from childbirth and bond with their infants without sacrificing their income or job security [33].

Though most of these advocacy efforts are conducted in offline and online settings such as news reports, website and blog reports, academic workshops, conferences, and medical settings, Twitter is one of the most common platforms to promote these advocacy programs. However, there is limited research on how advocacy groups utilize the platform to create awareness of maternal mortality issues in the United States. The existing research has focused on quantitative analyses. For instance, one study investigated the feasibility of using Twitter to identify public discourse on maternal health among Black women across the United States. The study revealed that Twitter is a valuable source for providing a snapshot of relevant topics to guide Black maternal health advocacy efforts [6]. There is a need to examine how advocacy groups use Twitter to raise awareness about US maternal mortality in ways that quantitative research cannot fully explore. This study uses qualitative content analysis to analyze solutions to maternal mortality discussed by maternal health advocacy groups on Twitter. Specifically, we sought to answer the following question: What maternal mortality solutions did maternal health advocacy groups discuss on Twitter?

## 2. Materials and Methods

We used qualitative content analysis to analyze tweets from 20 maternal health advocacy organizations on Twitter. We used qualitative content analysis instead of textual machine-learning analysis because the focus was not on emotional, sentimental analysis, or word count but on categorizing, interpreting, and analyzing themes generated from the data and their meanings [34]. The traditional qualitative content analysis allowed us to detect quotes and attributions suitable to support the themes.

### 2.1. Data Collection

We collected data in three stages: The first involved identifying maternal health advocacy organizations on Twitter. We used the keywords “maternal health advocacy”, “maternal health awareness”, and “maternal health mortality crisis” to search for maternal health advocacy Twitter accounts. We used these keywords to ascertain that we collected tweets only related to the maternal health crisis and advocacies and not to other maternal health issues, such as core medical terminologies in maternal health. We also found some Twitter accounts from peers’ retweets and following lists. Furthermore, we used Google Search to find maternal health advocacy organizations and looked them up on Twitter. We only selected the accounts that indicated their primary goals were to advocate for maternal health care in the United States. All accounts were based in the United States and had at least 400 followers. Further details of the Twitter accounts are located in Table 1.

In the second stage, with assistance from Vicinitas’s online Twitter history tracking platform [35], we downloaded all the tweets the selected groups had posted from January 2020 to December 2022. Given that the COVID-19 pandemic contributed to the high maternal mortality rates and its awareness, we wanted to capture public conversations during and towards the end of the pandemic. We downloaded and combined 26,746 tweets from the 20 Twitter accounts.

The third stage involved data filtering. Retweets were removed because we were only interested in analyzing the original tweets of the advocacy groups. From this large sample (*n* = 10,312), we selected the 250 most liked tweets from each Twitter account (*n* = 4500). Researchers assert that likes and comments predict users’ engagement and participation in social media posts [36,37]. In a similar study, the authors selected 200 tweets for each user [38]. However, we chose 250 per group to broaden our chances of finding relevant solutions discussed across the groups. The final sample tweets also were frequently retweeted and had high levels of engagement from Twitter users. We coded the 3021 tweets related to maternal health solutions from this sample. The other 2771 were primarily promotional tweets stating maternal mortality’s causes and implications.

### 2.2. Qualitative Content Analysis

Our research focused mainly on categorizing the maternal health solutions discussed in the tweets and visualizing the frequency of each solution’s occurrence. We defined maternal health solutions as tweets that concentrate on what should be done to reduce maternal mortality in the United States. We employed thematic content analysis using NVivo(14, Lumivero, Denver, USA), a software program commonly used for qualitative data analysis. Thematic content analysis (TCS) unpacks data sources, presents underlying themes, and adds nuance and cultural context to messages across groups [39,40]. Open coding, a thematic analysis method, was adopted to allow the data to speak for itself and uncover themes the researchers might not have considered before. The first two authors independently coded 10% (*n* = 302) of the data to identify and agree on the most important and relevant concepts, themes, subthemes, and patterns emerging from the data. When an agreement was reached and the coding process understood by all, we created a codebook (Table A1) and then divided the remaining sample so that each coder coded half of the remaining tweets. Overall, four main themes emerged from the data. To visualize and compare the occurrence of themes, including keywords, we used the NVivo software and Seaborn, a Python data visualization library based on Matplotib.

## 3. Results

**Figure 1:**
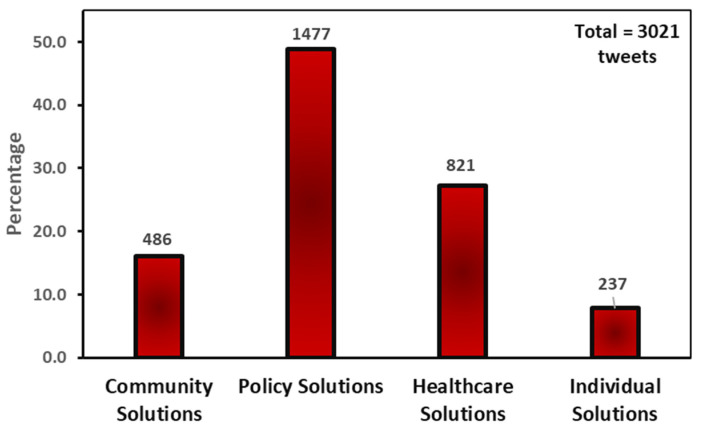
Main themes frequency.

Four main themes emerged from the study: policy, healthcare, community, and individual solutions. As Figure 1 shows, policy and healthcare solutions were the most tweeted, whereas community and individual solution tweets were the least tweeted.

### 3.1. Policy Solutions

**Figure 2:**
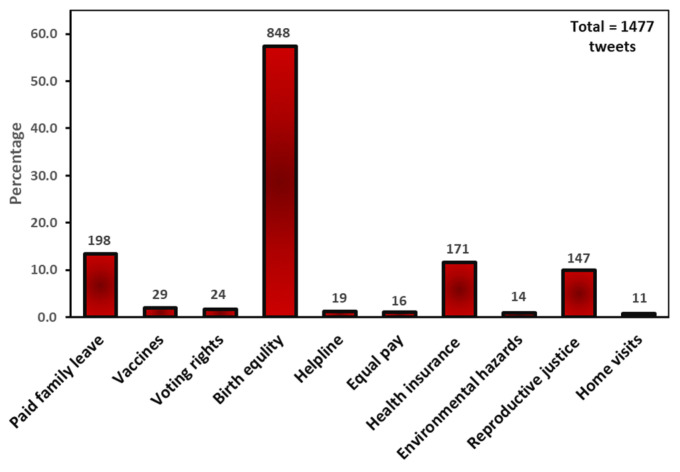
Policy solution tweets frequency.

Policy solutions were the most tweeted solutions, and nine policies were identified. Figure 2 shows that the most tweeted policies were the birth equity policies (57.4%), the paid family leave bill (13.4%), Medicaid expansion (11.6%), and the reproductive justice bill (10%). The least tweeted policies were focused on vaccines (2.0%), voting rights (1.6%), helpline (1.3%), equal pay (1.1%), environmental justice (0.9%), and home visits (0.7%). The birth equity policies were proposed to bridge the health disparities mainly affecting Black, immigrant, homeless, trans, incarcerated, and veteran mothers. Details of these policies are in Table 2.

The paid family leave tweets addressed the need to pass bills protecting parents’ jobs while nurturing their newborns, especially for Black and Brown women. For example, Every Mom Counts tweeted, “No one should have to choose between their family and a paycheck, their health and their work”. The tweets about Medicaid expansion called for Medicaid coverage of doula services and extending Medicaid coverage for pregnant women from six months postpartum to 12 months. These expansions could enable low-income pregnant and postpartum women to access comprehensive medical and behavioral health services for effective prevention and treatment.

Tweets about reproductive justice deplored the implications of the abortion ban on Black and Brown women who already face higher risks of health complications or death related to pregnancy or childbirth. The tweets argued that the federal government should deregulate abortion to reduce maternal mortality rates in the United States. Fewer tweets referenced the need for citizens to go out and vote for policymakers who would give attention to policies that would improve maternal health in the United States. Tweets mentioning helplines focused on addressing maternal mental health and reducing maternal suicide by establishing helplines across rural communities in the States. In contrast, tweets about equal pay centered on the effects of gender pay gaps in the United States and how Black and Brown mothers would have healthier pregnancies if they received the same salaries as men. The environmental justice tweets lamented that pregnant women who live in polluted areas are at higher risks of having a preterm birth, low birth weight, stillbirth, or infants born with congenital heart disease. These tweets called on Congress to pass bold legislation to address the impact of the climate change crisis on pregnant people in the United States. Lastly, the home visit tweets advocated for the passage of a law providing home visitation programs for newborns and mothers, particularly in rural areas and maternity care deserts.

### 3.2. Healthcare Solutions

**Figure 3:**
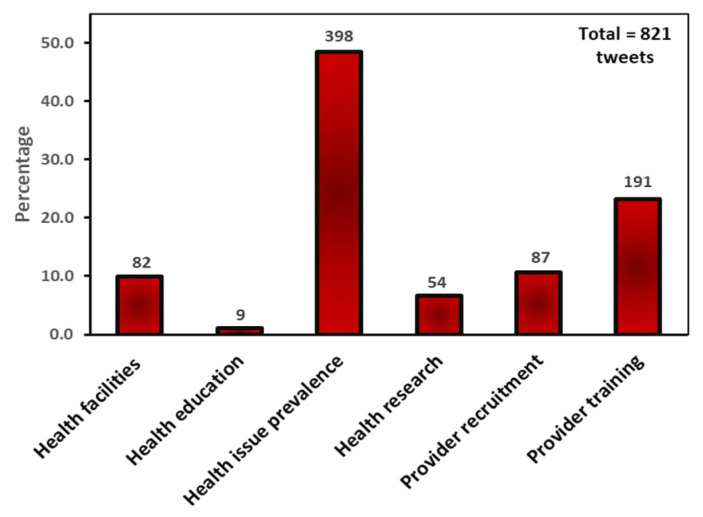
Healthcare solutions tweets frequency.

As Figure 3 shows, healthcare-focused solution tweets were categorized into six subthemes: health issue prevalence (48.5%), provider training (23.3%), provider recruitment (10.6%), health facilities (10%), health research (6.6%) and health education (1.1%). Tweets about health issue prevalence provided information about the most common complications of pregnancy and childbirth and the need for healthcare professionals to address them. The most tweeted health issue was maternal mental health, including postpartum depression, maternal suicide, substance abuse, and perinatal OCD. Advocacy groups such as 2020 Mom urged healthcare professionals to screen postpartum and pregnant women for mental health disorders during hospital visits while shedding stigma and creating awareness of the prevalence and signs of mental health disorders. Other identified health issues that needed to be addressed included high cesarean section rates, low breastfeeding rates, the effects of COVID-19 on pregnant women, high blood pressure, chronic conditions, preeclampsia, homicide, and sepsis.

The provider training tweets addressed the medical negligence affecting Black and minority mothers and the failure of healthcare providers to listen to, believe, and respond to mothers’ health-related concerns because they want to prevent mothers from having maternal complications. Several tweets in this category called for robust anti-racist training to sensitize healthcare providers about biased practices, beliefs, structural and institutional racism, and the policies perpetuating them. The tweets also encouraged providers to have humility, build trust and respect with pregnant and postpartum patients, focus on the mothers, and integrate them as partners rather than viewing them only as patients. Hashtags such as #sayhername, #hearher, #listentoher, and #believerher were prominent in these tweets. 

Provider recruitment tweets discussed recruiting Black physicians, midwives, and nurses to work in US hospitals. The Black Maternal Health Caucus noted that the mortality rates of Black women shrunk by 39% and 58% when Black physicians took charge of births. The tweets stated that racial disparities in healthcare could increase poor maternal health outcomes; thus, investing in Black birth workers can save lives, improve health, strengthen the health systems, and increase patient satisfaction. Tweets categorized as relating to health facilities discussed the need for healthcare systems to harness digital innovations and develop digital apps and programs that could provide evidence-based prenatal and postpartum care, help pregnant women to track their maternal health journeys, and reduce racial disparities. These tweets also promoted some available pregnancy apps such as Safe Delivery App, Together for Her Health app, Online Emotional Wellness Tool, Believer Her app, and a new e-learning program from Health & Human Services called Culturally and Linguistically Appropriate Services (CLAS) for maternal healthcare providers and students. One tweet asserted that telemedicine could act as a bridge between vulnerable pregnant women and their providers. 

Tweets categorized as health research reflected diverse perspectives and expertise in maternal health research—involving art and social science perspectives and centering more women in maternal health research. For instance, MotherLab tweeted, “Black women researchers bring a combination of lived experiences and research training together to apply love, social justice, and applied research principles to the field of maternal health”. In addition, the tweets encouraged improvement in data collection, quality measurement, evidence-based solutions, and research funding. Health education was the least often tweeted, but it advocated for more inclusive and in-depth patient education related to breastfeeding, prenatal care, caregiving, and childbirth education for pregnant women and their families.

### 3.3. Community Solutions

**Figure 4:**
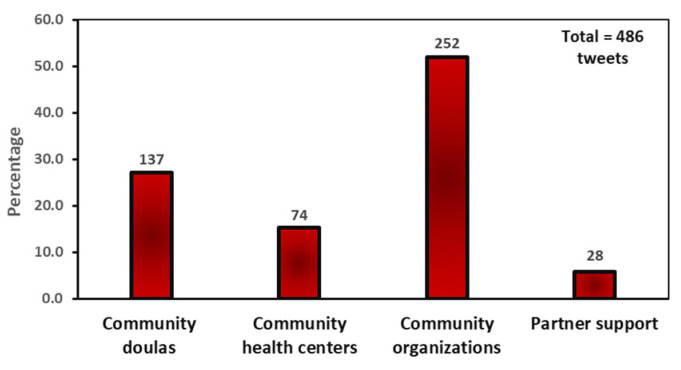
Community solution tweets frequency.

As shown in Figure 4, tweets focused on community solutions were categorized into four subthemes: funding community organizations (51.9%), hiring community doulas (27.2%), building community health centers (15.2%), and enhancing partner support (5.8%). Tweets about community organizations addressed the need for government and businesses to fund community organizations that work to improve the health of mothers and babies and create awareness about health disparities and health issues affecting pregnant women and their families. 

Community doulas—nonclinical birth workers—were promoted as a solution for Black and Brown pregnant women living in maternity deserts and underserved communities. Several tweets pointed out that community doulas do not replace providers. Instead, they are trained to provide pregnant women with the physical, emotional, and informational resources they need throughout the birth process. In addition, a doula’s presence can be incredibly calming and empowering to pregnant women and mothers who have experienced medical racism.

Tweets about community health centers addressed the frequent closures of hospitals in many communities during the COVID-19 pandemic. The tweets communicated that building more community health centers is mandatory for pregnant women in rural communities to have high-quality, respectful, and equitable care. Partner support tweets recognized the role of partners, husbands, or fathers in maternal health. The tweets reflected that for many women, their partners are first responders to their perinatal stress and significantly support them in seeking professional help.

### 3.4. Individual Solutions

**Figure 5:**
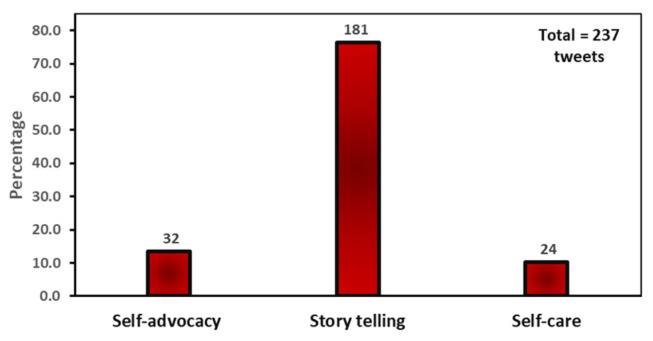
Individual solution tweets frequency.

As shown in Figure 5, individual solution tweets were categorized into three groups: storytelling (76.4%), self-advocacy (13.5%), and self-care (10.1%). Storytelling tweets urged mothers to use their voices and stories to demand equitable care because telling stories about their experiences sparks actions that can improve healthcare and support other mothers and families in communities. MoMMa’s Voice stated, “By standing up and sharing your experience, you give mothers and babies a chance to have better and safer outcomes”. Self-advocacy tweets encouraged mothers to assert themselves by speaking up and seeking help when they have medical concerns, especially during a high-risk pregnancy. The self-care tweets promoted the need for mothers to plan for healthy pregnancies by engaging in radical self-care—for example, working fewer hours, exercising frequently, eating healthy foods, utilizing peer support apps and podcasts, seeking information about their health risks, and having a deep, harmonic relationship between their mind and body.

## 4. Discussion

This study used qualitative content analysis to examine the Twitter accounts of 20 maternal health advocacy organizations to understand the solutions they promote for reducing maternal mortality in the United States. The findings revealed that the most tweeted solutions were related to changes in policy and the healthcare system, whereas the least commonly referenced solutions were related to community and individual efforts. Given the power of social media, especially Twitter, to create awareness about health issues and set the agenda for public discourse, these findings have several implications. 

Research has shown that health policies have the potential to address health disparities, improve access to healthcare, and prevent and manage diseases more rapidly than other interventions [41,42]. Studies also indicate that the more the media talk about an issue, the more attention and curiosity are drawn to these problems [43,44]. In addition, consistent coverage of an issue in the media alerts the government to take remedial actions [45]. As this study’s results indicate, advocacy groups primarily tweeted about policy solutions, with greater attention given to birth equity policies, paid family leave, and Medicaid expansion. This may explain why in November 2021, the government passed the Protecting Moms Who Served Act, which addresses the maternal health crisis among women veterans by helping improve care at VA facilities, particularly among women of color [46]. The measure was one of the 12 bills in the Black Maternal Health Momnibus Act introduced in March 2020 by the Black Maternal Health Caucus. The Momnibus Act initially aimed to improve health outcomes for Black birthing people but extended to pregnant and postpartum women, climate-change-related risks, and maternal vaccination. In March 2021, the Biden administration also signed the American Rescue Plan into law, which allowed states to extend Medicaid coverage for birthing people to 12 months postpartum [47]. Although our study cannot determine whether these Twitter advocacies were influential in passing these bills, the data show that these measures were prominently and frequently mentioned in advocacy group tweets, suggesting that they may have contributed to the discourse that led Congress to recognize their importance. Not all the advocated bills have been passed, but there is the potential that consistent tweeting of the relevance of these policies could transform them into laws. 

The remaining 11 Momnibus bills are stalled in the Senate amidst ongoing negotiations [48]. To make progress on these bills, advocacy groups might be better off focusing on promoting these policies at the local/community and state levels. Reports indicate that Congress generally pays little attention to public opinion [49]. This means that advocacy focusing too much on getting Congress to enact maternal health-supportive policies might be a waste of effort. Advocacy groups are much more likely to respond to the concerns of business, financial, and political elites. This would not mean ignoring the primary goals of these national-level policies but promoting them as local or state policies first. Then, if enough states pass such policies, Congress is more likely to enact something similar.

Previous studies have shown that Black women are more likely to experience poor communication during perinatal and postpartum care encounters [50,51]. The advocated healthcare solution tweets, particularly those related to provider recruitment and provider training, can also potentially eradicate or at least reduce systemic racism and medical negligence in the US healthcare systems. Health professionals exposed to these tweets might experience feelings of empathy and humility and decide to take action to equip themselves with relevant culturally sensitive knowledge and skills to better care for their pregnant and postpartum patients. In addition, the Twitter discussion could encourage health system administrators to offer or require cross-cultural communication training for health professionals in their hospitals and clinics. Given the power of social media to influence users, managers of healthcare systems could also be encouraged to diversify their workforce by recruiting more Black women who have lived experiences and are well-trained to provide care. 

Community doulas have been proven to help women feel relaxed and confident during the birthing process [52,53]. This study’s findings also discussed the need to recruit doulas for improved maternal health care. For pregnant women and providers, Twitter users unaware of the potential of community doulas in combating medical negligence and maternal mortality, particularly for minority women, the tweets by advocacy groups serve as an opportunity for them to learn about what doulas can provide. The results of this study also reflect the need for governments to build more community health centers for pregnant women living in maternity deserts and lacking access to comprehensive care. Furthermore, this study discloses the value of funding community organizations such as nonprofits and churches that provide resources and evidence-based maternal health information and advocate local policies to improve the health of mothers and babies. These days, when people have become isolated and alone, especially since the pandemic, this solution is badly needed. 

Because individual efforts to manage health can be overpowered by factors people cannot control, such as socioeconomic and environmental issues, it seems reasonable that the individual solutions were least often mentioned in advocacy group tweets. Nevertheless, several tweets called on mothers and grandmothers to use their voices to tell their stories and advocate for themselves. This could help policymakers—especially at the community and state levels—understand the crucial need to pass legislation to provide more maternal health support. Thus, users who are mothers or relatives of mothers could be inspired to use their platforms to join in the advocacy for maternal health improvements. Overall, this study shows that policy, community, healthcare, and individual solutions are core solutions several maternal health organizations believe would reduce the high rates of maternal mortality rates in the US. It is worthwhile for health researchers and experts in the US to factor these solutions into their maternal health interventions. Findings from this study could also serve other populations and communities experiencing high maternal mortality rates. It is important for health communication researchers to continue to explore discussions of maternal health issues on social media, especially Twitter. Social media conversations and advocacies could be a source of fundamental ideas for improving maternal health in the United States and globally.

## 5. Scope and Limitations of Study

Maternal mortality rates in the US remain among the highest among all developed countries. This study was a qualitative content analysis of maternal health solutions most tweeted by maternal health organizations in the United States. There were a few limitations. First, qualitative content analysis was used for this study. Though the method provides a rich understanding of the generated themes, future researchers could use textual machine-learning analysis to study sentimental analysis and word counts of most tweeted maternal health issues. This study was also neutral in selecting maternal health advocacy organizations to analyze. We did not target any particular racial group because many health tweets from all advocacy groups highlighted the racial disparities in maternal mortality rates. However, future studies could target organizations that focus mainly on a particular racial group to ascertain differences in the solutions they discuss.

Similarly, this study examined only the solutions to maternal mortality. In the future, researchers could take on a broader perspective and explore other issues discussed, such as the causes and consequences of maternal mortality in the US. Researchers could also examine profiles of people who support or oppose these discussed solutions to have a deeper understanding of audience perspectives on the possibilities of these solutions in improving maternal health in the United States. Finally, future research can explore the specific roles of policymakers, community doulas, family members, and community organizations in tackling maternal health issues in the United States. 

## 6. Conclusions

The result of this study provides valuable insights into the priorities of maternal health advocates. The findings that the tweets focused on policy, healthcare, community, and individual solutions highlight the multifaceted nature of the issue and the need for comprehensive approaches to address it. By advocating for policy changes, improving healthcare access and quality, mobilizing communities, and promoting individual solutions such as self-care and storytelling, maternal health advocates are working toward reducing maternal mortality rates and improving maternal health outcomes. Twitter and other social media platforms have been valuable tools in driving advocacy efforts, building connections, and amplifying the voices of marginalized communities [52,53]. As we continue to work toward solutions for maternal mortality in the United States, it is essential to listen to the voices of advocates and prioritize evidence-based solutions that address the complex and intersectional nature of these critical public health issues.

## Figures and Tables

**Table 1:** Twitter characteristics of maternal health advocacy organizations.

	Name of Organization	Location	No. of Followers	Year Joined
1	Merck for Mothers	Rahway, NJ, USA	21,800	October 2014
2	Maternal Health Learning and Innovation Center	North Carolina, USA	1263	March 2020
3	Maternal Mental Health NOW	Los Angeles, CA, USA	2346	February 2012
4	MoMMA’s Voices	Melbourne, FL, USA	1248	October 2018
5	MOTHER Lab	Massachusetts, USA	1531	September 2020
6	Dr. Shalon’s Maternal Action Project	Atlanta, GA, USA	811	April 2020
7	National Partnership for Women & Families	Washington, DC, USA	38,600	June 2010
8	Maternal Health Hub	Washington, DC, USA	569	February 2020
9	Partnership for Maternal and Child Health	Newark, NJ, USA	70,000	September 2007
10	March of Dimes	Arlington, VA, USA	64,000	August 2007
11	HealthConnect One	Chicago, IL, USA	2636	January 2013
13	Every Mother Counts	New York, NY, USA	26,300	April 2011
14	Equitable Maternal Health Coalition	Arlington, VA, USA	1225	June 2020
15	Black Maternal Health Caucus	Washington, DC, USA	9695	September 2019
16	National Birth Equity Collaborative	New Orleans, LA, USA	9420	February 2015
17	Black Mamas Matter Alliance	Atlanta, GA, USA	25,300	September 2016
18	AWHONN	Washington, DC, USA	12,700	June 2009
19	2020 Mom	Los Angeles, CA, USA	2929	October 2014
20	Equity Before Birth	Durham, NC, USA	416	October 2020

**Table 2:** Examples of tweeted policies.

Tweeted Policies	Explanations
Momnibus Act	The Black Maternal Health Momnibus Act, sponsored by Rep. Lauren Underwood, comprises 12 individual bills sponsored by Health Maternal Health Caucus Members. The legislation proposes to protect moms who served, invest in social determinants of health that influence maternal health outcomes, fund community-based organizations working to improve maternal health outcomes, grow and diversify the perinatal workforce, improve data collection, support maternal mental health conditions, improve maternal healthcare for incarcerated moms, invest in digital tools such as telehealth, promote innovative payment models, invest in addressing the unique risks and effects of COVID-19 during and after pregnancy, invest in initiatives to reduce the impact of climate change risks on moms and babies, and promote maternal vaccinations to protect the health and safety of moms and babies. So far, only one of the bills—Protecting Moms Who Served Act—has been signed into law.
The Maternal Action Plan	This plan was created by the Centers for Medicare & Medicaid Services (CMS) to improve maternal health outcomes and reduce disparities during pregnancy, childbirth, and postpartum. The plan aims to enhance maternity delivery to enrollees in Medicare, Medicare, Medicaid, the Children’s Health Insurance Program (CHIP), and the federal insurance marketplaces.
The American Rescue Plan	The American Rescue Plan of 2021 gives states a new option to extend Medicaid postpartum coverage from 60 days to 12 months. Under the new law, postpartum coverage can also be lengthened under the CHIP program. Some states have passed this bill, while others have not.
The Data Save Moms Act	This legislation, sponsored by Rep. Sharice Davids, would require the Federal Communications Commission (FCC) to identify areas where high rates of poor maternal health outcomes overlap with a lack of access to broadband services to pinpoint where telehealth services can be most effective, which is still in negotiation.
Family and Medical Insurance Leave (FAMILY) Act	This legislation, sponsored by Rep. Rosa DeLauro and Sen. Kirsten Gillibrand, would guarantee access to paid leave and meet the needs of pregnant people, caregivers, and families. Currently, families are only entitled to 12 weeks of unpaid leave, which is still in negotiation.
Healthy Families Acts	This legislation, sponsored by Sen. Patty Murray, would allow working people to take smaller increments of leave, in hours or days, to care for themselves and their families, which is still in negotiation.
PUMP for Nursing Mothers Act	This legislation would ensure that all working parents who need to express breastmilk during the workday have access to reasonable break time and private, non-bathroom space—passed on 29 December 2022.
Pregnant Workers Fairness Act (PWFA)	This legislation would address pregnancy discrimination by requiring that employers provide reasonable accommodations to allow pregnant workers to continue working safely without risking their health or their pregnancies. It has been passed and will go into effect on 27 June 2023.
Maternal, Infant, and Early Childhood Home Visiting Reauthorization Act of 2022	This legislation would reauthorize the Maternal, Infant, and Early Childhood Home Visiting (MIECHV) program, slated to expire on 16 December 2022. MIECHV funds the state to support evidence-based family and child home-visiting programs in high-risk communities. This bill was passed on 2 December 2022.

## Data Availability

The data available for this study are available from the corresponding author.

## Appendix A

**Table A1:** Codebook for Maternal Solution Tweets.

Main Themes	Meaning	Examples
Policy Solutions	Policy Solution was coded when the tweets mentioned acts or policies that have been passed or needed to be passed to reduce maternal mortality in the United States.	Women have carried us through this pandemic, at home and on the frontlines. They have lost over 5MM jobs and women of color had the greatest losses. We must pass #PaidLeaveForAll to protect working families, save jobs and create new growth.
Healthcare Solutions	Healthcare Solution was coded when the tweets talked about what hospitals and providers should do to reduce maternal mortality in the United States.	Harnessing digital innovations and putting the maternal needs of women at the center = critical to improving maternal health outcomes.
Community Solutions	Community Solution was coded when the tweets mentioned communities’ donations, fundraising, volunteer works, and educational events meant for mothers and their families.	It’s World Doula Week! We’re grateful for all doulas do to advocate for the physiological, social, emotional, and psychological health of women, newborns, and families in birth and postpartum.
Individual Solutions	Individual solution was coded when the tweets were targeted at mothers and pregnant women and the actions they should take to have a safer and healthier pregnancy journey.	This month, we are amplifying the power of storytelling in the treatment and recovery of perinatal health disorders. You are not alone in your journey and sharing your story is a way to let others know they are not alone too.
